# Coinfection outcome in an opportunistic pathogen depends on the inter-strain interactions

**DOI:** 10.1186/s12862-017-0922-2

**Published:** 2017-03-14

**Authors:** Hanna Kinnula, Johanna Mappes, Lotta-Riina Sundberg

**Affiliations:** Department of Biological and Environmental Science (and Nanoscience Center), Jyvaskyla, Finland

**Keywords:** *Flavobacterium columnare*, Genotype, Competition, Cooperation, Inhibition, Virulence, Zebra fish

## Abstract

**Background:**

In nature, organisms are commonly coinfected by two or more parasite strains, which has been shown to influence disease virulence. Yet, the effects of coinfections of environmental opportunistic pathogens on disease outcome are still poorly known, although as host-generalists they are highly likely to participate in coinfections. We asked whether coinfection with conspecific opportunistic strains leads to changes in virulence, and if these changes are associated with bacterial growth or interference competition. We infected zebra fish (*Danio rerio*) with three geographically and/or temporally distant environmental opportunist *Flavobacterium columnare* strains in single and in coinfection. Growth of the strains was studied in single and in co-cultures in liquid medium, and interference competition (growth-inhibiting ability) on agar.

**Results:**

The individual strains differed in their virulence, growth and ability for interference competition. Number of coinfecting strains significantly influenced the virulence of infection, with three-strain coinfection differing from the two-strain and single infections. Differences in virulence seemed to associate with the identity of the coinfecting bacterial strains, and their pairwise interactions. This indicates that benefits of competitive ability (production of growth-inhibiting compounds) for virulence are highest when multiple strains co-occur, whereas the high virulence in coinfection may be independent from in vitro bacterial growth.

**Conclusions:**

Intraspecific competition can lead to plastic increase in virulence, likely caused by faster utilization of host resources stimulated by the competitive interactions between the strains. However, disease outcome depends both on the characteristics of individual strains and their interactions. Our results highlight the importance of strain interactions in disease dynamics in environments where various pathogen genotypes co-occur.

**Electronic supplementary material:**

The online version of this article (doi:10.1186/s12862-017-0922-2) contains supplementary material, which is available to authorized users.

## Background

While recent studies have demonstrated that a single host is often infected by a multitude of pathogen strains or species [1–11], empirical investigations of the effects of coinfections on disease dynamics and virulence (i.e., damage caused to the host) are still limited (but see e.g., [11–13]), and their importance for many diseases is still unknown [14]. Coinfections affect both pathogen transmission in a population and pathogen virulence, thus influencing disease dynamics [15]. Coinfections can also influence host immune responses [3, 4] and effectiveness of disease control (see e.g., [16]). For these reasons, they are likely to contribute to virulence evolution, although the understanding on these evolutionary consequences is limited [15].

The disease outcome in a coinfected host results from complex interactions between the host and the coinfecting strains. Whether the inter-strain interactions are neutral, cooperative or competitive, depends on the genetic relatedness of the strains (see [17–19]). Closely related pathogens are likely to cooperate and exploit their hosts economically in order to maximize their transmission, while distantly related pathogens are more likely to compete, leading to increased virulence and decreased transmission due to facilitated host death [19, 20]. High relatedness can lead to low virulence if the cooperation of pathogens leads to prudent host exploitation [20] or to high virulence if the cooperation leads to faster growth [21]. Similarly, low relatedness may cause high virulence via increased growth and resource use [17, 18] or reduced virulence, if the competing pathogen strains produce toxins targeted to kill the competitor [22]. In the latter case the pathogen strains experience a cost, when the energy is allocated to toxin production instead of growth or virulence.

There are three types of competition that coinfecting pathogens may encounter: resource competition, interference competition or apparent competition [4]. Resource competition takes place when the coinfecting conspecific strains have overlapping resource requirements. In other words, the strain that processes nutrients more efficiently might outgrow the competing strain in nutrient-limiting conditions [23]. In interference competition the coinfecting strain secretes molecules that harm its competitor, e.g., bacteriocins [24, 25]. Bacteriocins have a narrow activity range and thus interference competition is often more prevalent between conspecific than distantly related strains [26]. The third form of competition is so-called apparent competition that results from indirect exclusion of the coinfecting strains by host immune response stimulated by growth of one strain that acts on both of the competing strains [4, 14, 27].

Coinfections may be especially important for the ecology and evolution of opportunistic pathogens that are often able to persist in and transmit from the environment [28]. The opportunists with a wide host range have a higher likelihood to find potential hosts than host-specialists [28, 29]. They are therefore more likely to be involved in coinfections. *Flavobacterium columnare* (Bacteroidetes) is a globally important host generalist fish pathogen with an opportunistic lifestyle and an ability to transmit environmentally [30–33]. The bacterium causes columnaris disease in cultured freshwater fish, with typical symptoms including gill necrosis and skin erosion [34]. Genetically different *F. columnare* strains with variable levels of virulence and growth rates are known to co-occur at fish farms [31, 35]. Furthermore, the virulent strains were found to have an increased ability for interference competition, and it is likely that these strains have a competitive advantage in the aquaculture environment [35]. However, a direct test of whether coinfections influence the virulence of *F. columnare* is missing. Here, we study the effect of two- and three-strain coinfection on virulence of *F. columnare* using zebra fish (*Danio rerio*) as a model host. We ask whether the inter-strain interactions are related to changes in virulence and growth through competition.

## Results

### Virulence in zebra fish hosts

**Fig. 1:**
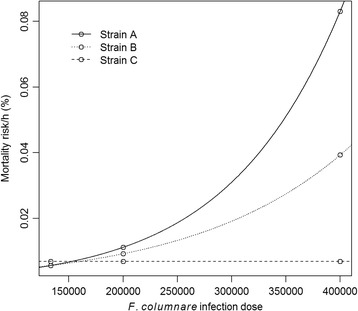
The estimated mortality risk per hour of zebra fish (*Danio rerio*) infected with strain A (*continuous line*), strain B (*dotted line*) and strain C (*dashed line*) of *Flavobacterium columnare* with doses of 1. $\overline{3}$ ×10^5^, 2 × 10^5^ or 4 × 10^5^ CFU ml^−1^

We found a significant interaction between bacterial dose and strain identity on their effect on the host mortality risk (*p* < 0.001, Fig. 1, Tables 1 and 2). The risk of infection of the host increased along with the dose when the fish were infected with strain A or strain B. In more detail, strain A was the most virulent, while strain B expressed intermediate level of virulence. Strain C caused zero mortality in zebra fish hosts independent of the used infection dose.

**Table 1:** The effect of bacterial dose and host species on the mortality risk of the hosts in the virulence experiment(N.S. = not significant)

Source	Estimate	Std. Error	*P*-value
(Intercept)^a^	−6.569	3.182^−1^	<0.001
Dose	1.042^−5^	1.073^−6^	<0.001
StrainB	4.039^−1^	4.536^−1^	N.S.
StrainC	1.599	4.614^−1^	<0.001
Dose: StrainB	−2.998^−6^	1.526^−6^	<0.01
Dose: StrainC	−1.042^−5^	1.640^−6^	<0.001

^a^Intercept includes the effects of strain A

**Table 2:** The significance and test values of the bacterial dose and strain on the mortality risk of zebra fish

Source	Df	Deviance	Residual deviance	*P*-value
Dose	1,151	52.217	162.940	**<0.001**
Strain	2,149	44.326	118.614	**<0.001**
Dose: Strain	2,147	42.340	76.274	**<0.001**

Significant *P* values are denoted in bold

**Fig. 2:**
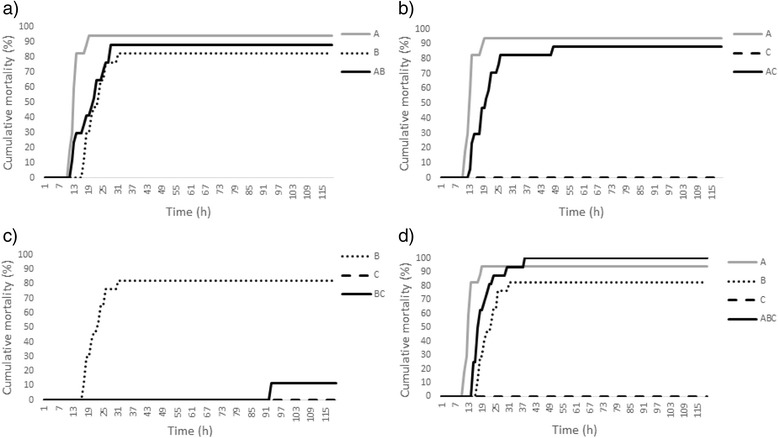
Cumulative mortality of zebra fish (*Danio rerio*) as a function of time in the virulence experiment with single-strain and multiple-strain infections: coinfection of strains **a**) A and B, **b**) A and C, **c**) B and C, and **d**) A, B and C. The treatment groups are presented in the legend

When we compared the single- and coinfection treatments, we found that mortality of the fish differed significantly between the treatment groups (Cox regression Wald = 39.6, N = 118, df = 6, *p* < 0.001, Fig. 2, Table 3). The number of strains influenced the virulence (Wald = 9.47, *N* = 118, df = 2, *p* < 0.01), with the average longevity in the three-strain coinfections being 19.6 h (S.E.M. 1.6), while the longevity was 59.8 h in the two-strain coinfections and 59.3 h in the single infections (S.E.M. 6.9 and 7.2, respectively). In pairwise comparisons (with log rank Mantel-Cox with corrections for multiple testing), virulence of the single and two-strain coinfections did not statistically differ, but the other combinations did (single vs. three-strain coinfection: *χ*2 = 6.97, *p* < 0.05, two-strain vs. three-strain coinfections: *χ*2 = 11.306, *p* < 0.05).

**Table 3:** Pairwise differences in virulence of *Flavobacterium columnare* in single and in coinfections. **(**Pairwise Kaplan-Meier survival analysis with log rank Mantel-Cox)

Treatment	A		B		C		A + B		A + C		B + C	
	*χ*2	Sig.	*χ*2	Sig.	*χ*2	Sig.	*χ*2	Sig.	*χ*2	Sig.	*χ*2	Sig.
B	15.286	**<0.001**										
C	33.464	**<0.001**	25.016	**<0.001**								
A + B	7.179	.007	.621	.431	28.772	**<0.001**						
A + C	9.479	**.002**	1.021	.312	28.877	**<0.001**	.007	.936				
B + C	31.823	**<0.001**	21.675	**<0.001**	2.063	.151	26.103	**<0.001**	26.206	**<0.001**		
A + B + C	6.551	.010	5.183	.023	38.611	**<0.001**	.930	.335	1.136	.287	38.611	**<0.001**

After correction for multiple testing, *p*-values below 0.0024 can be considered significant (denoted in bold)

The coinfection treatments where strain A was involved were the most virulent independent of the strains included in coinfection, indicating that strain A may have a dominant role in coinfections in this study (Table 3). Respectively, when strain A was not involved in the coinfection treatment, the treatment resulted in very low virulence. The bacterial samples taken from the gill tissue of the infected and moribund fish were positive for *F. columnare*, whereas the bacterial samples taken from the gill tissue of the control fish were negative. The pure cultures made from the infection solutions revealed that colonies formed by strains A and B expressed the virulent rhizoid morphotype, but colonies of strain C represented the rough type, which has been linked with decreased virulence [36]. As the negative control fish were not infected, they were not included in the statistical analyses.

### Interference competition on agar

Strains B and C were able to inhibit the growth of strain A, and strain C was able to inhibit the growth of strain B (Table 4). Strain A did not inhibit the other strains in this study. When the strains were exposed to the sterile-filtered supernatant, no signs of inhibition were observed, indicating that growth inhibition in *F. columnare* could be dependent on a cell-cell contact.

**Table 4:** The inhibition profiles of *Flavobacterium columnare* strains A, B and C

	Donor			Donor (sterile-filtered)		
Recipient	A	B	C	A	B	C
A		2	2		0	0
B	0		1	0		0
C	0	0		0	0	

The number inside the cell stands for the frequency of inhibition in three independent experiments

### Growth in single-strain and co-cultures

First, in relation to the dose controls in the virulence experiment, the individual strains were cultured in vitro in three doses (33, 50 and 100%) to reveal the influence of the original bacterial dose on bacterial replication. The bacterial dose was found to have a significant effect on the maximum growth rate of the strains (measured as the maximum slope of the linearized growth curve) (F = 4.408, df = 2, 58, *p* < 0.05), and on the time when maximum yield (highest OD) was achieved (F = 22.528, df = 2, 58, *p* < 0.001 (Additional file 1: Figure S1). Bacterial strain included as a random factor in the analyses did not significantly influence any of the growth variables.

**Fig. 3:**
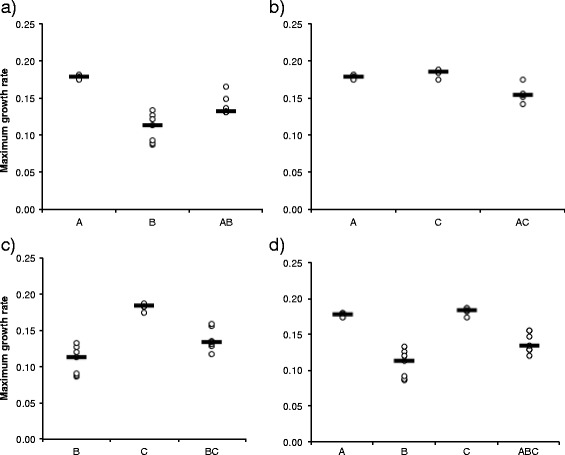
Scatterplot for maximum growth rate (with line denoting median) of *Flavobacterium columnare* in single and in co-culture. **a**) Strains A and B and their co-culture, **b**), strains A and C, **c**) B and C, and **d**) A, B and C

**Fig. 4:**
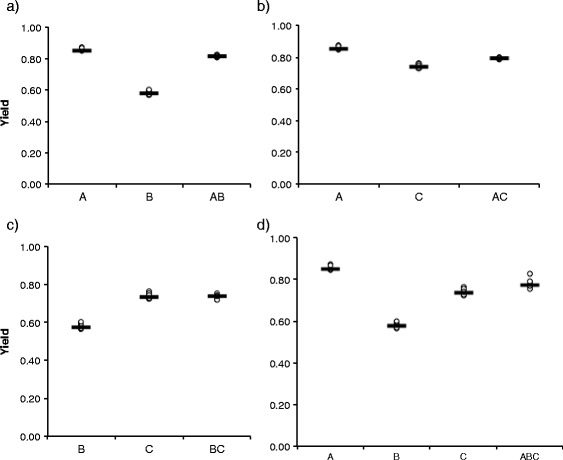
Scatterplot for yield (with line denoting median) of *Flavobacterium columnare* in single and in co-culture. **a**) Strains A and B and their 1:1 co-culture, **b**), strains A and C, **c**) B and C, and **d**) A, B and C

**Fig. 5:**
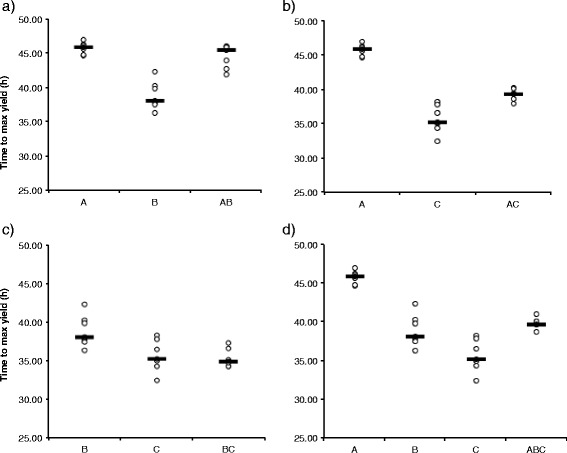
Scatterplot for time to reach maximal yield (with line denoting median) of *Flavobacterium columnare* in single and in co-culture. **a**) Strains A and B and their 1:1 co-culture, **b**), strains A and C, **c**) B and C, and **d**) A, B and C

Secondly, to understand the increased virulence in the three-strain coinfection, we studied the effect of co-culture on bacterial growth. Single or co-culture did not have overall significant effects on the bacterial growth parameters (Additional file 2: Table S1). However, the pairwise comparisons in the non-parametric ANOVA revealed some significant differences between individual strains and co-culture combinations (Table 5, Figs. 3, 4 and 5).

**Table 5:** Statistically significant results of relevant pairwise comparisons of all single (strains A, B, C) and co-cultures (combinations AB, AC, BC and ABC) in Kruskal-Wallis One-Way ANOVA

	Maximum growth rate		Yield		Time to max yield	
Culture pair	Kruskal-Wallis	*p*	Kruskal-Wallis	*p*	Kruskal-Wallis	*p*
A-B	33.857	<0.001	42.000	<0.001		
B-C	−39.143	<0.001				
C-A			30.571	0.001	34.643	<0.001
B-BC	−26.429	0.011				
B-AB			34.000	<0.001		
B-ABC			23.714	0.04		
C-BC					−36.786	<0.001
C-ABC	−26.571	0.011

## Discussion

Environmentally transmitted bacterial pathogens are likely to cause both single and coinfections in their hosts, which may lead to plastic changes in virulence, but also to evolutionary change under long time scales. Coinfections can lead to increased or decreased pathogen virulence, depending on the nature of the interactions between the coinfecting bacterial strains [22]. We examined how different strains of an opportunistic fish pathogen, *F. columnare*, interact with each other in culture, and how coinfection with these strains affects the disease virulence (measured as zebra fish mortality). We found that virulence was significantly influenced by the number of the coinfecting strains, infection with three strains leading to higher fish mortality than infection with one or two strains. Generally, increased virulence can be related to the growth-stimulating mutualistic [37] or competitive interactions between the coinfecting strains [20]. However, we found no unambiguous evidence of enhanced bacterial growth in co-culture in this study, indicating that the higher virulence in three-strain coinfection results from other factors. One possibility is that the host immune system has difficulties to cope with a heterogeneous pathogen inoculum compared to a single-strain infection (see e.g., [38]).

Here, strain A had the highest virulence in single infections. Similarly, the presence of strain A in coinfections resulted in highly virulent infections, indicating a dominating effect by this strain during infection, even with lower bacterial doses. Relative doses of the coinfecting strains and relative virulence impact the outcome of within-host competition [12, 13]. Based on our results, the effect of dose on bacterial virulence in single infections increases exponentially in *F. columnare*, the lower doses not being sufficient to cause mortality in zebra fish. Thus, the single strains could not have been responsible for the high host mortality in the three-strain coinfection treatment as their individual doses were only one third of the total infection dose. The high virulence in the three-strain coinfection treatments must therefore result from other factors, such as a plastic response to within-host competition (via gene expression), or factors related to the host immune system.

The faster growth of the competing strains resulting in increased pathogen replication (resource competition) and the inability of the host immune system to handle the heterogeneous simultaneous multiple infection are commonly associated with the increased virulence in coinfections [20]. However, we did not observe systematic differences in bacterial in vitro growth rate between single and co-cultures. Furthermore, the elevated virulence due to immune response is often related to coinfection by different parasite species or types [39, 40]. Therefore, the strain interactions most likely have a central role in the elevated virulence in the three-strain coinfection of zebra fish in this study. Indeed, rapid evolution of plastic changes in parasite virulence in response to coinfection has been documented in bacteriophages [41], indicating that the variability in the infection outcome arises from the genetic interactions between the coinfecting partners.

Relatedness of the coinfecting strains has a fundamental role in strain interactions and virulence of coinfection, and interference competition (targeted to hamper the growth of competing strains or species) is common in bacteria [24, 42–44]. *Flavobacterium columnare* is a genetically homogeneous species [45], and according to the MLSA analysis, the genotypes C and E used in this study are closely related [46]. However, the genomic content outside the housekeeping genes often differs substantially between bacterial strains, suggesting genetic variability in these strains isolated originally from different fish farms in different years. This is important, because the production of growth-inhibiting toxins and immunity against them are genetically linked [47]. This is clearly seen as the capacity of the bacterial strains in distinguishing between “self” and “non-self” competitors in the inhibition assay. Therefore, considering interference competition without genome sequencing, MLSA grouping does not give a complete picture on the genome-level relatedness of the strains, and the exact level of relatedness between the strains used in this study cannot be resolved without whole genome sequencing.

In general, production of growth-inhibiting toxins is expected to be costly for the producer strain, and to be traded off with growth [22, 48]. In some cases, release of toxins may even require cell death, as in *E. coli* [49, 50]. Our earlier studies indicated that the growth inhibiting toxin-production and tolerance may be linked with increased virulence in *F. columnare* [35]. However, coinfections by genetically diverse strains producing these toxins are predicted to reduce the population growth rate and consequently the virulence of the infection [22]. Yet, similarly to the previous study [35], we did not find any indications that toxin production would be traded off with growth in *F. columnare*. Interestingly, however, the virulence of two-strain coinfection was directly linked with virulence of the individual strains, resulting in lower virulence in the two-strain coinfections than in the three-strain coinfection. Moreover, the inhibitory compounds produced by the less virulent strains C and B against the highly virulent strain A could explain the reduced virulence observed in these coinfections. However, it seems that decreasing the relatedness of the coinfecting partners (by increasing the number of strains) makes interference competition most beneficial, resulting in higher virulence of the three-strain coinfection as observed here.

Our study demonstrates that pairwise interactions between coinfecting strains are important for disease virulence. Similar findings have been observed in several studies using different host-parasite systems, e.g., rodent malaria parasite *Plasmodium chabaudi* [51], snail-infecting schistosome *Schistosoma mansoni* [38], baculoviruses [52] and bacteria (see [48, 53, 54]). Therefore, perhaps not surprisingly, the genetic interactions between the coinfecting parasites may also extend across species [55], challenging the reliability of epidemiological predictions based on single infections. As the coinfections [56, 57] and environmental pressures maintain the diversity of the pathogen populations and induce rapid changes in pathogen traits [41], coinfections are among the main drivers for variance in disease epidemiology and evolution.

## Conclusions

Intraspecific competition can lead to increased virulence but the virulence of coinfection depends on the interactions of the strains involved in the coinfection. As the likelihood of coinfections by environmentally transmitted opportunistic pathogens is high, the genetic composition of the coinfecting population can lead to variable infection outcomes. This can significantly impact the outcomes of infections. Furthermore, in opportunists not restricted by the transmission-virulence trade-off, coinfections may select for the most virulent pathogen strains.

## Methods

### Bacterial strains and culture conditions

We used three previously isolated *F. columnare* strains obtained from two different fish farms in different years to study the effect of coinfection on virulence of opportunistic bacteria (Table 6). The bacteria were originally isolated using standard culture methods on Shieh medium supplemented with tobramycin [58] and stored frozen in −80 °C in a stock containing 10% glycerol and 10% fetal calf serum. The strains A and C were genetically characterized in earlier studies using multilocus sequence analysis (MLSA) [46] and strain B using automated ribosomal intergenic spacer analysis (ARISA) [35]. The genetic clustering produced by MLSA method is shown to be comparable with the ARISA method [46]. Before the experiments, the strains were grown in modified Shieh medium [59] at 26 °C under constant shaking (150 rpm) for 48 h and enriched (1:10) overnight to an early log phase.

**Table 6:** The strains used in the study

Bacterial strain	Code	Isolation source	Year of isolation	Fish farm	Genetic group
B259	A	Rearing tank water	2009	Fish farm A in Central Finland	C
B350	B	Fish farm outlet water	2010	Fish farm A in Central Finland	E
B424	C	Atlantic salmon, *Salmo salar*	2007	Fish farm B in Northern Finland	C

Genetic grouping is based on ARISA genotyping (Kunttu et al. 2012, Sundberg et al. 2016). The fish farm A produces mainly rainbow trout (*Onconrhynchus mykiss*) fingerlings for food production, and farm B salmonid fingerlings (salmon *Salmo salar*, trout *Salmo trutta*) for stocking purposes

### Virulence in zebra fish hosts

The virulence of the *F. columnare* strains was studied in apparently disease-free zebra fish (average weight 0.384 g, standard deviation 0.114) that were obtained from the Core Facilities (COFA) and research services of Tampere (University of Tampere, Finland). Prior to the experimental challenge, the zebra fish were maintained in aerated borehole water in 250-liter aquaria at 25 °C and fed once a day (1% of body weight) with commercial feed (Special Diet Services).

The experimental set-up consisted of seven treatment groups (all strains alone, and in two- and three-strain combinations), six dose control groups of individual strains (50% and 33. $\overline{3}$% of the single infection dose) and a negative control group (see Additional file 3: Table S2). The fish were individually challenged in 0.5 liter of aerated borehole water using a previously optimized continuous infection method (see [60]).

The OD of the overnight-grown bacterial culture was measured at 570 nm and the corresponding bacterial density in colony forming units (CFU) was calculated according to our previously fitted standard curve (results not shown). The infection method and bacterial dose used were optimized in preliminary experiments. To infect the fish, the volume of bacterial solution was adjusted to 600 μl of modified Shieh medium (Song et al. [59]) and applied directly into the aquaria to reach a total infection dose 4 × 10^5^ CFU ml^−1^ in the water. This means that in the coinfection treatments, the proportion of each strain was either a 50% or 33. $\overline{3}$% of the total dose (i.e., 2 × 10^5^ or 1. $\overline{3}$ ×10^5^ CFU ml^−1^, respectively, with corresponding control treatments with each strain, see Table 7). In each of the seven treatment groups and in the dose control groups, 17 replicate hosts were infected. In addition, 10 negative control fish per treatment were sham-exposed to sterile Shieh medium. Thus, in total, 231 fish were used in the experiment. After experimental infections, pure cultures of the infection solutions were spread on Shieh agar plates to confirm that the bacteria were expressing rhizoid morphotype typical to virulent *F. columnare* (see [36]).

**Table 7:** Model selection based on Akaike information criteria (AIC) of the effect of bacterial dose on virulence

Model	AIC	df	*P*
Strain + Dose + Strain: Dose	410.50	6	
Strain + Dose	448.86	4	<0.001
Dose	489.20	2	<0.001

+ describes the main effects and colon the interactions

The best fit model estimating mortality risk of the zebra fish within time is underlined. *P* value indicates the significance of the term removed from the higher model

After bacterial infection, the aquaria were randomized on the shelves in the experimental room to reduce the effect of possible temperature differences, and the fish were monitored for disease symptoms and mortality for 5 days at 1 h intervals, the first 48 h day and night. When the disease-induced mortality ceased, the observation points were decreased to four checks per day. The water temperature was maintained at 25.7–25.9 °C throughout the experiment. The fatally moribund fish, that did not react to external stimuli, were removed from the experiment, given terminal anesthesia using 0.01% MS-222 (Sigma, in ethanol buffered with sodium bicarbonate), and decapitated to confirm death. The fish surviving until the endpoint of the experiment (including the control fish) were euthanized in the end of the experiment. Gill samples were taken from every fish and cultured on 1% Shieh agar plates supplemented with tobramycin (1 μg ml^−1^). The yellow colonies with a rhizoid morphology were considered as a sign of the columnaris disease.

### Interference competition on agar

The interference competition between *F. columnare* strains was studied reciprocally using a standard double layer method, as described previously [35]. Three hundred microliters (300 μl) of fresh overnight-grown “recipient” bacterial culture were adjusted to OD of 0.29 (A570 with VICTOR X Multilabel Plate Reader, Perkin-Elmer, USA), mixed with three milliliters (3 ml) of soft Shieh agar (0.7%) tempered to 47 °C, and poured on Shieh agar plates (1%). One milliliter (1 ml) of the remaining bacterial culture was centrifuged for 3 min at full speed (13 000 G) to separate the bacterial cells from the culture solution. Five hundred microliters (500 μl) of supernatant was taken into a new Eppendorf tube to avoid bacteria dissolving from the pellet. Ten microliters (10 μl) of this “donor” supernatant were spotted on the surface of the agar containing the bacteria. To evaluate whether growth-inhibiting molecules were secreted in the cell-free supernatant, one milliliter (1 ml) of supernatant was sterile-filtered (0.2 μm PES filter, VWR) and 10 microliters (10 μl) of the filtrate were spotted on the agar plates. The plates were incubated for 48 h at room temperature, after which they were checked for growth inhibition (clear zones with no bacterial growth) caused by the “donor” strains and ranked as 0 = no inhibition, 1 = inhibition. Three independent replicates were done.

### Growth in single-strain and co-cultures

For growth measurements the fresh overnight-grown bacterial cultures were adjusted to OD of 0.15–0.20 in A570. Thirty microliters (30 μl) of each bacterial strain or two- or three-strain combinations was transferred onto a new BioScreen plate (100-well-plate) and supplemented with three hundred microliters (300 μl) of sterile Shieh culture media. The proportion of one strain in two-strain co-culture was 15 μl and 10 μl in three-strain co-culture. To evaluate how the inoculum size affects bacterial growth, half and third “doses” were included in the experiment as controls, where a half (15 μl) or two thirds (20 μl) of the bacterial inoculum was replaced with sterile water. The measurements were done in seven replicates per treatment in Shieh medium at 25 °C. The growth data was recorded for 96 h at 5 min intervals (absorbance at 420–580 nm, wide band option) using Bioscreen™ spectrophotometer (Growth Curves Ltd., Helsinki, Finland).

The growth parameters were calculated from raw data by using a MATLAB script (created by Tarmo Ketola, see [35]), where the maximal growth rate of the pathogen can be found from log_2_-transformed data by fitting linear regressions on 20 time-point sliding windows. The highest linear slope (the exponential growth is linearized due to the log transformation) found in sliding windows is equal to the maximal growth rate of the bacteria. The yield was measured as a maximal average optical density over 20 time point’s sliding window in the raw data.

### Statistical analysis

Mortality data from the dose controls was analysed with generalized linear model (GLMM) for binomial distribution to evaluate the effect of dose on zebra fish mortality risk. The mortality risk of host per hour was modelled as a function of dose (1. $\overline{3}$ ×10^5^, 2 × 10^5^ or 4 × 10^5^ CFU ml^−1^, included as a continuous covariate), and bacterial strain (strain A, strain B and strain C). The independent variables and their interaction were included in the model to find the best model to explain fish mortality. The model was simplified based on Akaike information criteria using a backward stepwise procedure (Table 7). The analysis was conducted with the software R 2.15.2 and the glm function (R Development Core Team 2011). When interpreting the effects of the terms included in the model, a significance level of 0.05 or less was used.

To evaluate the influence of coinfection on *F. columnare* virulence, host mortality data of the single-strain and multiple-strain infections was analyzed with Cox regression using treatment and number of strains as categorical covariates. To compare virulence of the individual treatments, pairwise comparisons of the treatment groups and number of infecting strains were conducted using non-parametric Kaplan-Meier survival analysis with log rank Mantel-Cox test. The significance level in the pairwise tests was manually corrected (by dividing by number of tests performed) for multiple testing, and *p*-values below 0.0024 were considered significant. The analyses were performed with IBM SPSS Statistics version 22.0 (IBM).

The growth traits (maximum growth rate, yield, time to maximum yield) of the bacterial populations in single- and co-cultures (A, B, C, A + B, A + C, B + C and A + B + C) were analyzed with mixed model with REML option in SPSS. Single/co-culture was used as a fixed factor, and culture identity (non-diluted individual cultures) was included as a random factor. We also used Kruskal-Wallis oneway ANOVA with multiple comparisons implemented in SPSS to determine the pairwise differences in growth characteristics between bacterial strains and co-cultures. Figures for growth parameter comparisons were drawn according to [61].

## Additional files

Additional file 1: Figure S1. Bacterial densities (mean ± S.E.M.) of dose controls of strains A, B and C of *Flavobacterium columnare* in the growth experiment. The average optical density of the cultures is on the Y-axis, and culture time on the X-axis. (DOC 65 kb)
Additional file 2: Table S1. Effect of the treatment (single or coinfection) on the growth parameters of *Flavobacterium columnare. (DOC 29 kb)*

Additional file 3: Table S2. The treatment and control groups in *Flavobacterium columnare* infection experiment. (DOC 70 kb)
