# Supplementary material for: Coinfection outcome in an opportunistic pathogen depends on the inter-strain interactions
Source: BMC Evol Biol. 2017 Mar 14;17:77. doi: 10.1186/s12862-017-0922-2 (PMC5348763; doi:10.1186/s12862-017-0922-2)
Supplement: Additional file 1: Figure S1. — Bacterial densities (mean ± S.E.M.) of dose controls of strains A, B and C of Flavobacterium columnare in the growth experiment. The average optical density of the cultures is on the Y-axis, and culture time on the X-axis. (DOC 65 kb) [file 12862_2017_922_MOESM1_ESM.doc]

**
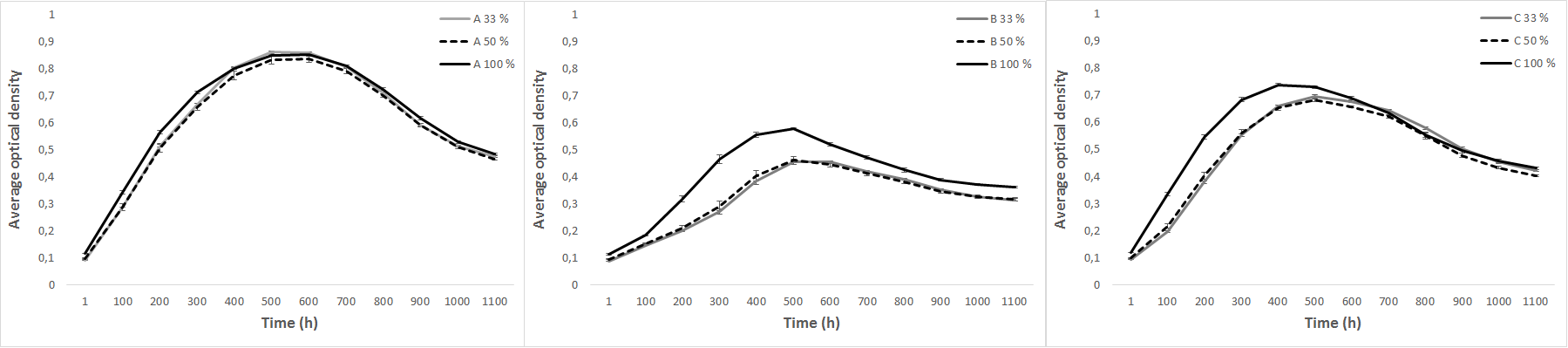
**

**Supplementary Figure 1.** Bacterial densities (mean ±S.E.M.) of dose controls of strains A, B and C of *Flavobacterium columnare* in the growth experiment. The average optical density of the cultures is on the Y-axis, and culture time on the X-axis.
