# Supplementary material for: Coinfection outcome in an opportunistic pathogen depends on the inter-strain interactions
Source: BMC Evol Biol. 2017 Mar 14;17:77. doi: 10.1186/s12862-017-0922-2 (PMC5348763; doi:10.1186/s12862-017-0922-2)
Supplement: Additional file 2: Table S1. — Effect of the treatment (single or coinfection) on the growth parameters of Flavobacterium columnare. (DOC 29 kb) [file 12862_2017_922_MOESM2_ESM.doc]

***Supplementary Table 1.*** *Effect of the treatment (single or coinfection) on the growth parameters of Flavobacterium columnare*.

|  | **Single vs. co-culture** | | |  |  | **Culture ID** |  |  |  |  |  |
| --- | --- | --- | --- | --- | --- | --- | --- | --- | --- | --- | --- |
|  | F | df1 | df2 | p-value |  | Estimate | std error | Z | p-value | 95%CI lower | 95%CI upper |
| **Maximum growth rate** | 0.473 | 1 | 5 | 0.522 |  | 0.000712 | 0.000464 | 1.534 | 0.125 | 0.000199 | 0.002556 |
| **Yield** | 0.604 | 1 | 5 | 0.472 |  | 0.008129 | 4.047910^-5^ | 1.576 | 0.115 | 0.002344 | 0.028194 |
| **Time to maximum yield (h)** | 0.011 | 1 | 5 | 0.920 |  | 19.0319 | 12.22382 | 1.557 | 0.119 | 5.404125 | 67.018974 |
