# Supplementary material for: Coinfection outcome in an opportunistic pathogen depends on the inter-strain interactions
Source: BMC Evol Biol. 2017 Mar 14;17:77. doi: 10.1186/s12862-017-0922-2 (PMC5348763; doi:10.1186/s12862-017-0922-2)
Supplement: Additional file 3: Table S2. — The treatment and control groups in Flavobacterium columnare infection experiment. (DOC 70 kb) [file 12862_2017_922_MOESM3_ESM.doc]

**Additional file Table 2.** The treatment and control groups in *Flavobacterium columnare* infection experiment.

| **Treatment** | **Infection dose (CFU ml^-1^)** | **Shieh volume (µl)** | **Number of replicates** |
| --- | --- | --- | --- |
| **A** | 4×10^5^ | 600 | 17 |
| **B** | 4×10^5^ | 600 | 17 |
| **C** | 4×10^5^ | 600 | 17 |
| **A+B** | 4×10^5^ (2×10^5^ × 2) | 600 | 17 |
| **A+C** | 4×10^5^ (2×10^5^ × 2) | 600 | 17 |
| **B+C** | 4×10^5^ (2×10^5^ × 2) | 600 | 17 |
| **A+B+C** | 4×10^5^ (1.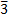×10^5^ × 3) | 600 | 17 |
| **0.5 A (dose control)** | 2×10^5^ | 300 | 17 |
| **0.5 B (dose control)** | 2×10^5^ | 300 | 17 |
| **0.5 C (dose control)** | 2×10^5^ | 300 | 17 |
| **0.33 A (dose control)** | 1.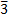×10^5^ | 200 | 17 |
| **0.33 B (dose control)** | 1.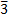×10^5^ | 200 | 17 |
| **0.33 C (dose control)** | 1.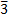×10^5^ | 200 | 17 |
| **Negative control** | None | 600 | 10 |
